# Physicochemical Heuristics for Identifying High Fidelity, Near-Native Structural Models of Peptide/MHC Complexes

**DOI:** 10.3389/fimmu.2022.887759

**Published:** 2022-04-25

**Authors:** Grant L. J. Keller, Laura I. Weiss, Brian M. Baker

**Affiliations:** Department of Chemistry & Biochemistry and the Harper Cancer Research Institute, University of Notre Dame, Notre Dame, IN, United States

**Keywords:** peptide, major histocompatibility complex, neoantigen, structure, prediction, support vector machine

## Abstract

There is long-standing interest in accurately modeling the structural features of peptides bound and presented by class I MHC proteins. This interest has grown with the advent of rapid genome sequencing and the prospect of personalized, peptide-based cancer vaccines, as well as the development of molecular and cellular therapeutics based on T cell receptor recognition of peptide-MHC. However, while the speed and accessibility of peptide-MHC modeling has improved substantially over the years, improvements in accuracy have been modest. Accuracy is crucial in peptide-MHC modeling, as T cell receptors are highly sensitive to peptide conformation and capturing fine details is therefore necessary for useful models. Studying nonameric peptides presented by the common class I MHC protein HLA-A*02:01, here we addressed a key question common to modern modeling efforts: from a set of models (or decoys) generated through conformational sampling, which is best? We found that the common strategy of decoy selection by lowest energy can lead to substantial errors in predicted structures. We therefore adopted a data-driven approach and trained functions capable of predicting near native decoys with exceptionally high accuracy. Although our implementation is limited to nonamer/HLA-A*02:01 complexes, our results serve as an important proof of concept from which improvements can be made and, given the significance of HLA-A*02:01 and its preference for nonameric peptides, should have immediate utility in select immunotherapeutic and other efforts for which structural information would be advantageous.

## Introduction

Genomic instability can result in thousands of mutations within transformed cells (1). During normal housekeeping, peptide fragments containing mutations can be bound by class I major histocompatibility complex (MHC) proteins and presented extracellularly where they are surveilled by CD8+ T cells of the cellular immune system. However, while cellular immunity is generally capable of distinguishing between self and non-self, most mutant peptides are not recognized as non-self. This results in part from thymic selection, during which self-reactive T cells are culled, as well as various peripheral tolerance mechanisms which prevent T cell reactivity towards self antigens. Thus, mutant peptides bound to an MHC protein must overcome self-tolerance to constitute immunogenic “neoantigens” which induce T cell responses. Those mutant peptides that do overcome tolerance can lead to naturally occurring tumor immune responses or are candidates for therapeutic peptide-based vaccines (2–6). Identifying those mutated peptides which overcome tolerance and are most likely to initiate anti-tumor immunity, however, remains a significant challenge (7, 8)

Two non-exclusive mechanisms by which a mutant peptide can overcome self-tolerance are by enhancing peptide binding to MHC proteins and altering the structural and physical features presented to T cell receptors (TCRs). The former mechanism can lead to presentation of novel antigens, whereas the latter allows a mutated self antigen to be perceived as foreign. Enhanced binding to MHC can be addressed by various bioinformatics tools for estimating peptide-MHC binding affinities e.g., (9–12). Identifying changes in structural and physical features, however, is more challenging (13). Although some general structural features can normally be predicted with some confidence (for example, which peptide side chains are “up” and available for TCR contacts) (14), predicting fine details and changes that occur with mutations necessitates atomistic detail. The scope of the challenge is highlighted by recent results showing that neoantigen immunogenicity can be driven by subtle structural changes that occur away from the site of a mutation (15).

Recent work from our lab demonstrated the utility of peptide-MHC three-dimensional models in generating hypotheses for T cell immunogenicity with different peptides (7, 8, 16), assessing T cell receptor binding and specificity towards specific peptide-MHC complexes (17–19), and in predicting peptide immunogenicity (20). This work, and related work of others (21–29), demonstrates the value of structure in T cell-based therapeutic target prioritization, the development of personalized cancer vaccine approaches, and assessments of potential off-target epitopes. However, as opposed to general protein modeling, which has recently seen significant advances (30, 31), accurate modeling of peptides bound to MHC proteins, and class I MHC in particular, is particularly difficult, requiring high fidelity prediction of backbone and side chain positions, and for neoantigens, subtle structural changes that might emerge from mutations.

Modern structural modeling procedures commonly employ Monte Carlo or other sampling procedures to explore conformational space, resulting in the generation of numerous candidate models, typically referred to as decoys. A key question in these efforts is: which of these decoys is most representative of the actual structure? Although the lowest energy decoys are usually presumed to be the most accurate, work in other fields has shown that this is often not the case (32–35), an issue attributable in part to inaccuracies, tradeoffs, and simplifications in energy functions (35, 36). Here, we systematically examined the accuracy of peptide-MHC structural modeling procedures. We show that ranking and selecting decoys by energy is ineffective at reproducing known peptide-MHC structures. After identifying a more optimal sampling approach, we explored the applicability of system-specific functions based on structure-derived physicochemical properties for predicting deviations between a decoy and its actual structure. Trained on a large database of high-resolution structures of nonameric peptides presented by the class I MHC protein HLA-A*02:01, our functions significantly outperform decoy selection by energy, leading to substantially improved prediction of peptide structural features. Although currently restricted to nonamers presented by HLA-A*02:01, our improved methods suggest a way toward achieving the high fidelity needed for accurate identification of peptide-MHC structures *in silico* and may be of immediate use for evaluating nonamers presented by HLA-A*02:01.

## Methods

### Collection of Experimental Structures

Experimentally determined peptide-MHC structures used to evaluate modeling protocols were collected from the RCSB Protein Data Bank (PDB) using the REST API service (37). The submitted query specified β_2_-microglobulin (β_2_m), the MHC heavy chain allele HLA-A*02:01, and a chain of nine residues (the peptide). Structures with resolutions ≥ 3.0 Å and those containing proteins other than peptide-MHC complexes were excluded. This list was filtered for structures with unambiguous peptide electron density using Coot to inspect 2*F*_o_-*F*_c_ electron density maps (38). PDB ID 2GTW was excluded due to its register shifted decamer-like conformation (39). For structures with more than one molecule per asymmetric unit, only the first copy was used. The final set contained 103 high-resolution structures of non-redundant nonameric peptides bound to HLA-A*02:01. Of these, six structures (PDB IDs 5EU3, 6O4Z, 6PTB, 6VR5, 7KGO, and 7LG3) were excluded from training. These six, selected randomly from the structures not included in our previous study (20), were set aside to be used as a test set for evaluation of trained functions. All structures used are listed in **Table S1**. The same approach and criteria were used for selection of nonameric peptides bound to other class I MHC proteins.

### Structural Modeling of HLA-A*02:01-Presented Nonameric Peptides

Structural modeling of peptide-MHC complexes was conducted as previously described (20). Briefly, PyRosetta 4.0 (40, 41) was used with either the talaris2014 (42) or ref2015 (43) energy function as indicated, with starting coordinates from PDB 3QFD (44). As noted previously (20), when modeling performance was evaluated as a function of different template structures, 3QFD performed best, although the difference between templates was small (~0.2 Å heavy atom RMSD) and template choice is thus expected to have little influence on overall results. The template crystal structure was energy minimized using the FastRelax protocol in Rosetta (41, 45) with harmonic restraints of 0.02 kcal/mol, which we found optimally balanced reduction in energy with changes in atomic coordinates. Template peptide side chains were then replaced with those of the target peptide sequence. Next, all amino acid sidechains in the peptide and the MHC were repacked to energetically favorable rotamers using Rosetta PackRotamersMover (this was the extent of the “repack” protocol used as a negative control and assesses template-dependent bias). Peptide side chain and backbone atoms were then minimized using either 50 iterations of fragment insertion followed by simulated annealing CCD *via* LoopMover_Refine_CCD or neighbor-sensitive dihedral angle sampling followed by simulated annealing KIC with a maximum segment length of 12 *via* LoopMover_Refine_KIC. As structural modeling *via* Rosetta relies on Monte Carlo sampling, multiple independent models (decoys) were generated for each peptide-MHC. The number of decoys generated during modeling and used in specific analyses is noted where appropriate. “Rosetta energy” refers to the sum of weighted terms from the indicated Rosetta energy function for all residues of the peptide-MHC complex. Where indicated, “peptide energy” is only the sum of these for residues in the peptide chain.

### Scoring of Decoys for Regression and RMSD Calculations

For each residue of the peptide in each decoy generated, terms from the ref2015 energy function, total solvent accessible surface area (SASA), and hydrophobic solvent accessible surface area (hSASA) were calculated in Rosetta after modeling. SASA and hSASA calculations utilized a 1.4 Å radius probe. Data was filtered for regression to include only terms which exhibited non-zero variance for all decoys and were not specific to residue identity (e.g., tyrosine ring planarity was excluded). This resulted in 129 structural and energetic terms. For RMSD calculations, the target crystal structure was first superimposed on the heavy chain of the modeling template *via* the Cα atoms of residues 1-180. The root mean square deviation (RMSD) of atomic positions was then calculated between peptide residues only and is reported between either Cα or all non-hydrogen heavy atoms (HA).

### Regression Analysis of Full-Atom RMSD vs. Energetic Terms

Regression models (referred to as functions) for fitting heavy atom RMSDs between decoys and corresponding crystal structures to the 129 structural/energetic terms were calculated in R. Data was centered around the mean, scaled by term standard deviation, and randomly partitioned into a training set of 80% of cases for fitting functions and a test set of 20% for less biased evaluation of regression function performance, so training and test set had comparable RMSD distributions. Ordinary least squares (OLS) and partial least squares (PLS) functions were fit using the train function of the caret package and the pls package implementation in R. PLS functions were fit using 10 components after evaluation. Support vector machine regression (SVR) functions were trained using the e1071 implementation of SVR with an ϵ-insensitive loss function and either no kernel (linSVR) or a Gaussian radial basis function (radSVR) as the kernel function. The linSVR grid search covered regularization (C) values from 10^-8^ to 10^8^. For radSVR, values spanned 10^-4^ to 10^10^ for C, and 10^-11^ to 10^2^ for the width of the Gaussian radial basis function (γ). Final functions were trained with hyperparameter combinations that displayed the lowest root mean square error (RMSE) from this grid search. OLS, PLS, and SVR functions were subjected to 10-fold cross validation during grid search and training.

### Recombinant Protein Production, Crystallization, and Structure Determination

The purified complex of AVGSYVYSV with HLA-A*02:01 was generated by refolding heavy chain and β_2_m from bacterial inclusion bodies according to standard procedures (46), followed by purification using anion exchange and size-exclusion chromatography. Peptide was synthesized by Genscript at >90% purity. Crystals of the AVGSYVYSV complex were grown by hanging-drop vapor diffusion at 4°C in 15% PEG 3350 and 0.1 M MES, pH 6.5 from a concentration of 5.1 mg/mL diluted 1:1 with mother liquor. Crystals were harvested and cryoprotected in ~8% glycerol and ~92% mother liquor and then immediately frozen in liquid nitrogen. Data for the complex were collected at the NE-CAT 24ID-E beamline at the Advanced Photon Source at Argonne National Laboratories. Data integration and scaling were performed using the HKL2000 suite (47). Data reduction was performed in Aimless. The structure was solved by molecular replacement using Phaser in PHENIX (48), with PDB 3PWL with the peptide removed used as a search model (49). Multiple steps of restrained refinement were performed using PHENIX Refine (48). Evaluation of models and fitting to maps were performed using Coot (38). MolProbity was used to evaluate structures during and after refinement (50).

### Code Availability

Modeling scripts and regression functions have been deposited at the Zenodo repository, available at https://doi.org/10.5281/zenodo.6049929.

## Results

### Updated Structural Modeling Methods Improve Peptide-MHC Modeling Accuracy Yet Identifying Optimal Decoys Remains a Challenge

Previously, we developed a rapid approach for modeling class I peptide-MHC structures (20). Tested against a dataset of 53 high-resolution crystallographic structures of nonameric peptides presented by HLA-A*02:01, the most accurate structural models (hereafter referred to as decoys) exhibited average peptide heavy atom (HA) and α carbon (Cα) root mean square deviations (RMSD) from crystallographic coordinates of approximately 1.8 Å and 0.9 Å, respectively. While this performance was comparable to other published methods for modeling peptides bound to class I MHC proteins (23, 25, 26, 51–61), the RMSD range was large, with some final models deviating from crystal structures by more than 3 Å.

Structural modeling is dependent on both sampling algorithms and the energy (or score) functions used to evaluate conformers. Our previous approach used cyclic coordinate descent (CCD) loop modeling with the talaris2014 energy function. To gauge the effect of iterative changes to our approach, we evaluated replacing CCD with kinematic loop modeling (KIC). Both the CCD and KIC algorithms were developed to solve the inverse kinematics problem in robotics, although their application to protein loop modeling differs considerably: CCD in Rosetta relies on insertion of database-derived fragments followed by torsion angle adjustments, while KIC stochastically samples backbone torsions in a neighbor-dependent fashion with gradually decreasing weights on repulsive and Ramachandran components of the energy function. Although computationally more expensive, in direct comparisons KIC reliably sampled near-native loop conformations more frequently than CCD (62).

We also evaluated the energy function, replacing talaris2014 with the newer ref2015. The ref2015 energy function was the first Rosetta energy function to be parameterized on small molecule data in addition to polypeptides and statistical terms. It also incorporates more realistic electrostatic and solvation terms than previous functions and demonstrated improved performance over talaris2014 in ranking decoys and modeling loops (32). As a negative control, we evaluated talaris2014 with side chain repacking and no backbone dihedral modification (referred to as the “repack” protocol, which also controls for template bias).

**Figure 1:**
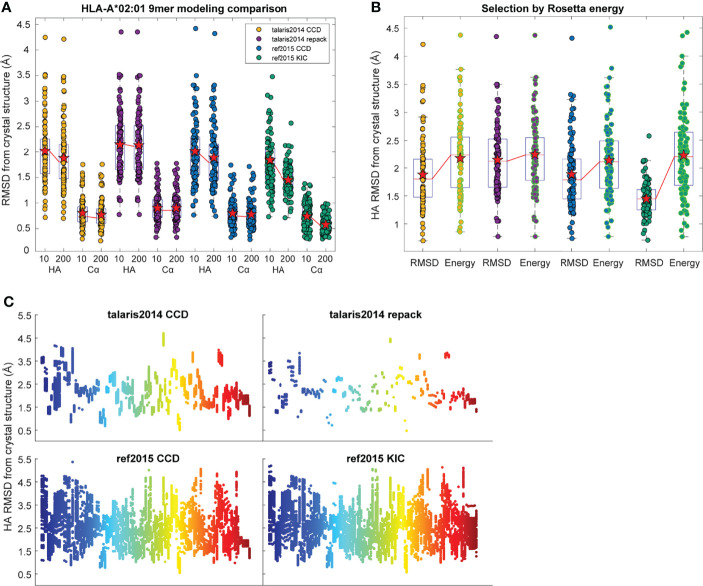
Performance of structural modeling protocols. **(A)** Distribution of peptide heavy atom (HA) and α carbon (Cα) RMSDs of the most accurate (lowest HA RMSD from crystal structure) decoys generated for 103 target peptide-MHC complexes when modeled by the four different protocols indicated. RMSDs were calculated for peptides only after superimposition of the HLA-A*02:01 peptide binding grooves (Cα atoms of heavy chain residues 1-180). Mean is indicated by a red star, boxes represent the first to third interquartile range, and horizontal lines show the median. The medians of 10 and 200 decoys are connected by red lines. Implementing ref2015 and KIC alone had little effect on accuracy, although decreased the variance in RMSD. Moving from 10 to 200 decoys resulted in significant improvement when using ref2015. **(B)** Distribution of peptide HA RMSDs of the most accurate of the 200 decoys from panel A (black outline) and the decoys with the lowest Rosetta energy (green outline). Mean is indicated by a red star; medians are connected by red lines. Colors for the modeling protocols are the same as in panel **(A)**. **(C)** Distribution of peptide HA RMSD from crystal structure (*y* axis) for 200 decoys of each target peptide-MHC (*x* axis), illustrating coverage of conformational space. The mean per-target variance, or degree of conformational sampling, of the ref2015 KIC protocol (0.27) was slightly higher than ref2015 CCD (0.22), and much higher than either talaris2014 CCD (0.017) or talaris2014 repack (0.0044). Points are colored across the spectrum for clarity.

In exploring these iterative changes, we focused exclusively on nonameric peptides presented by the human class I MHC allele HLA-A*02:01, as these dominate the corpus of experimentally determined human peptide-MHC structures. We expanded our dataset of high-resolution structures from 53 to 103, selecting those with resolutions <3.0 Å and unambiguous peptide electron density (**Table S1**). As previously performed, we initially generated only 10 decoys for each of the structures in our dataset. From these, we selected the actual best decoy, as measured by lowest peptide HA RMSD from the crystal structure (i.e., the most accurate structural model for the peptide as identified by comparison to the known structure). Note that in calculating RMSDs here and throughout, the Cα atoms of only the peptide binding grooves were superimposed and differences between peptide coordinates computed. Surprisingly, we found that implementing KIC and ref2015 resulted in little improvement in overall modeling accuracy as measured by average peptide Cα and HA RMSD, although both decreased the variance in RMSD (**Figure 1A**). To investigate how increased sampling impacted accuracy, we increased the number of decoys generated per protocol 20-fold to 200. The additional sampling did not lead to an improvement in the best decoys generated with the control talaris2014 repack protocol (again measuring lowest HA RMSD from crystal structure). There was however slight improvement in talaris2014 CCD HA RMSD, and the increased sampling significantly improved performance of ref2015 CCD and ref2015 KIC modeling as determined by a one-tailed Wilcoxon matched-pairs signed-rank test (**Figure 1A**; blue and green).

**Figure 2:**
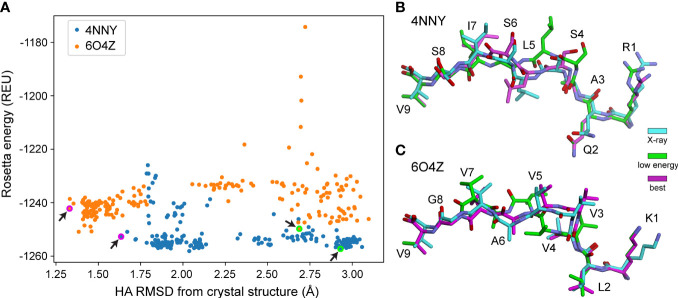
Structurally divergent decoys can have similarly low energies. **(A)** Rosetta energy vs. peptide HA RMSD from crystal structure for 200 decoys generated using ref2015 KIC for two peptide-MHC complexes (PDB IDs 4NNY and 6O4Z, colored as indicated). Decoys exhibit a wide range of RMSD values despite similarly low energies. Decoys shown in panels **(B, C)** are indicated with magenta/green circles and highlighted by the black arrows.**(B)** Visual comparison of two decoys for 4NNY. The crystal structure is colored cyan. The best decoy (lowest RMSD from structure) is magenta (-1253 REU, 1.63 Å HA RMSD, 0.63 Å Cα RMSD). A lower energy but poorer decoy is green (-1257 REU, 2.93 Å HA RMSD, 1.76 Å Cα RMSD). **(C)** Comparison for 6O4Z. The crystal structure is cyan, the best decoy (lowest RMSD from structure) is magenta (-1242 REU, 1.33 Å HA RMSD, 0.55 Å Cα RMSD), and a lower energy but poorer decoy is green (-1250 REU, 2.69 Å HA RMSD, 1.71 Å Cα RMSD).

We next asked if the greater sampling and updated energy function permitted better identification of optimal models based on lowest energy, the most frequently used criteria for selecting decoys when structures are unknown. We found though that despite improved modeling accuracy, the common problem of identifying the most optimal decoy remained, as the HA RMSD of decoys selected by lowest Rosetta energy did not differ significantly between the protocols used (**Figure 1B**). Thus, even with improved sampling that *can* generate better structural models, scoring by Rosetta energy is insufficient for identifying the *best* structural model for nonameric peptides presented by HLA-A*02:01. Two examples of how scoring by energy alone poorly accounted for peptide structural details are illustrated in **Figure 2**. Of the 200 decoys for each peptide-MHC target modeled with ref2015 KIC, most differ only by a few Rosetta energy units (REU) while spanning a HA RMSD range of nearly 3 Å relative to the crystal structure, as shown in **Figure 2A** for PDB IDs 4NNY (sequence RQASLSISV) and 6O4Z (sequence KLVVVAVGV). Two of the lowest energy decoys of 4NNY differ by only 4 REU (approximately 0.3%). However, while the conformation of one decoy is nearly identical to the crystallographic coordinates, with Cα/HA RMSD values of only 0.63/1.63 Å, the other, lower energy decoy exhibits poorer Cα/HA RMSD values of 1.76/2.93 Å. The conformation of this better scoring decoy deviates substantially from the crystal structure, fully exposing the position 5 side chain which in the structure serves as a secondary anchor and is thus buried in the MHC binding groove (**Figure 2B**). 6O4Z presents a similar case (**Figures 2A, C** **)**: the actual best decoy is again close to the crystal structure (Cα/HA RMSD = 0.55/1.33 Å), whereas a lower energy decoy differs substantially from the structure (Cα/HA RMSD = 1.71/2.69 Å), with the peptide backbone at positions 4-6 modeled incorrectly. Thus, despite generating more accurate structural models, improved sampling and an updated energy function do not necessarily translate into improved structural predictions if lowest energy is used to select optimal decoys as is commonly performed.

### Training Regression Functions to Predict the Deviations of Decoys From Actual Structures

To explore improved methodologies for identifying the most accurate structural model from a set of decoys, we took inspiration from two sources. First, some methods that predict peptide affinity for MHC proteins consider position-specific features determined by the different structures and chemistries of the pockets that line the MHC binding groove (9, 63). Second, efforts to improve protein structure prediction have explored using machine learning to selectively weight terms in energy functions, resulting in optimized decoy selections approaches trained for specific systems or tasks (28, 33, 34, 64–66). Accordingly, we explored regression approaches in which peptide position-dependent structural and energetic terms were differentially weighted to yield functions optimized for identifying near-native decoys of nonamers bound to HLA-A*02:01.

Training effective regression functions requires a range of responses. While the ref2015 energy function with either CCD or KIC performed comparably when considering the overall range of RMSD from target selected by energy (**Figure 1B**), the per-target variance of the ref2015 KIC protocol decoys was higher than the other protocols, indicating greater sampling (**Figure 1C**). We therefore standardized on this protocol and increased the number of decoys generated to 500 and used only these for training and selection.

We tested the efficacy of three types of regression functions in predicting decoy RMSD from crystal structure using energetic features of the peptide as described by the Rosetta ref2015 energy function. We first used ordinary least squares (OLS), the most common type of regression. OLS involves determining a multiplicative weight factor for each term in a linear equation and including an additive intercept term. An advantage of OLS is the weights are straightforward to interpret. After generating 500 decoys for each target peptide-MHC in **Table S1** (excluding the six used for validation as indicated in the table), we calculated the Cα and HA RMSD between the peptide chain of that decoy and the corresponding crystal structure. As we performed previously (20), energetic (from ref2015) and structural features were calculated for each residue of the peptide, describing interactions between peptide atoms, interactions of peptide atoms with surrounding MHC atoms, and interactions of peptide atoms with (implicit) solvent. As shown in **Table S2**, we included van der Waal’s interactions, hydrogen bonds, solvation potential, rotamer and backbone dihedral probabilities, and both hydrophobic and total peptide solvent accessible surface area. A linear function was trained to fit these sets of per-residue features for all decoys included in the training set to the HA peptide RMSD of that decoy from its crystal structure, such that the resulting function would predict RMSD from structure for any provided decoy. The resulting weights in the OLS function associated with per residue peptide features used as input are listed in **Figure S1**.

In addition to OLS, we trained a partial least squares (PLS) function to predict decoy RMSD from the same input features. Unlike OLS, PLS transforms features into a reduced dimensional space, similar to principal component analysis, which maximizes variance in response. We reasoned that a PLS function may account for relationships between input features, as PLS is less sensitive than OLS to feature collinearity while resulting in weights which can still be interpretable. We compared cross-validation RMSE and percent of variance explained in both input energy function features and RMSD from crystal structure upon inclusion of additional components, up to a maximum of 30. The improvement in explained variance and RMSE diminished past inclusion of 10 components, which we chose as the number of components to include in the final PLS function.

The third type of function we used to predict quality of structural models was support vector machine regression (SVR) (67). In the simplest case, SVR is similar to OLS regression with the addition of an “error insensitive” boundary term, where errors between predicted and actual response less than the boundary value are ignored, helping to reduce the influence of noise. SVR functions can be further extended *via* a kernel trick that increases dimensionality, allowing for better accounting for complex non-linear relationships. SVR functions have been employed in a number of biochemical and structural prediction problems (33, 68). We refer to a linear SVR function that does not employ a kernel trick as “linSVR” and a function which employed a Gaussian radial basis function as a kernel trick as “radSVR.”

Choice of SVR hyperparameters is critical, especially the regularization parameter *C* which represents a balance between error and function complexity (69). We conducted a logarithmically spaced massively parallel grid search to identify pareto-optimal hyperparameter combinations. The grid search to identify an optimal linSVR regularization hyperparameter presented a trough of error values (**Figure S2A**), from which the lowest RMSE value for *C* was selected. The radSVR grid search yielded an apparent local minimum (**Figure S2B**); however, it was not bounded on increasing values of γ, which corresponds to a wider Gaussian in the radial basis function and thus less influence on the decision boundaries of other support vectors. Due to overfitting concerns with high γ values, the search was not extended and the value for γ was selected that yielded the local minimum in RMSE.

To evaluate model bias, we compared the predictions for all functions to our training and test set to the actual RMSD and computed the cross-validation RMSE. The cross-validation RMSEs for OLS, PLS, linSVR, and radSVR were 0.37, 0.42, 0.14, and 0.22, respectively. The prediction frequencies for all models corresponded well with the actual RMSD, which did not implicate severe overfitting as an issue, although the radSVR function erroneously predicted a HA RMSD of 2.5 Å for some decoys regardless of actual RMSD, which was exacerbated in test set predictions and suggests some overfitting in this function (**Figure S3**).

### Trained Regression Functions Outperform Decoy Selection by Rosetta Energy

**Figure 3:**
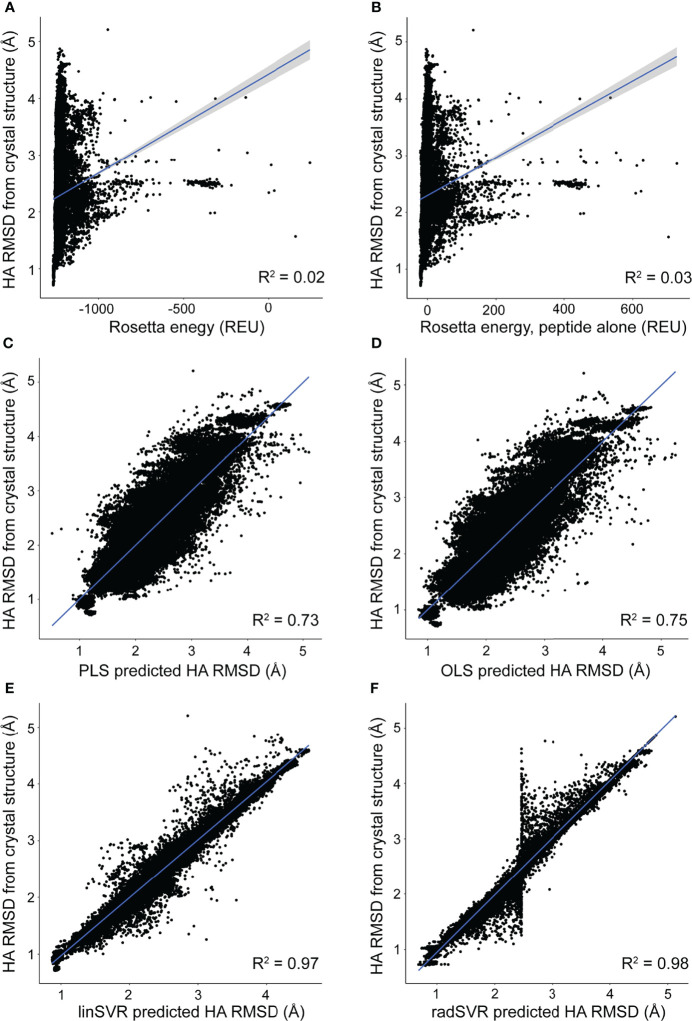
Trained functions better rank decoys in order of peptide RMSD from crystallographic structures. The peptide HA RMSDs for all 500 decoys for each of the crystallographic structures in **Table S1** (excluding the six test structures) were plotted against Rosetta energy of the peptide-MHC **(A)**, peptide alone **(B)**, or predicted HA RMSD from each of the trained functions **(C–F)**. There was no correlation between RMSD and Rosetta energy. In sharp contrast, predicted RMSD from trained functions correlate well with RMSD from structure **(C–F)**, with excellent correlations seen with the SVR functions **(E, F)**. A sharp split in the trend of the radSVR predictions around 2.5 Å likely reflects overfitting as discussed in the text (see also **Figure S3**). R^2^ values are indicated in each plot; 95% confidence intervals are shown, but only apparent in panels **(A, B)**.

The trained selection functions generated by PLS, OLS, linSVR, and radSVR were then used to compare predicted vs. actual HA RMSD from the crystal structure for the 500 decoys for each peptide-MHC in **Table S1**, excluding the six test structures. The trained OLS, PLS, and SVR functions significantly outperformed prediction by Rosetta energy for the complex or peptide alone, for which there was no correlation between predicted and actual RMSD (**Figures 3A, B** **)**. The trained functions showed good correlations, with the linSVR and radSVR functions showing superior performance compared to OLS and PLS (**Figures 3C–F**). The erroneous HA RMSD values of 2.5 Å for the radSVR data seen in validation (**Figure S3**) were apparent in this analysis (**Figure 3F** **)**, which raised caution about this function.

**Figure 4:**
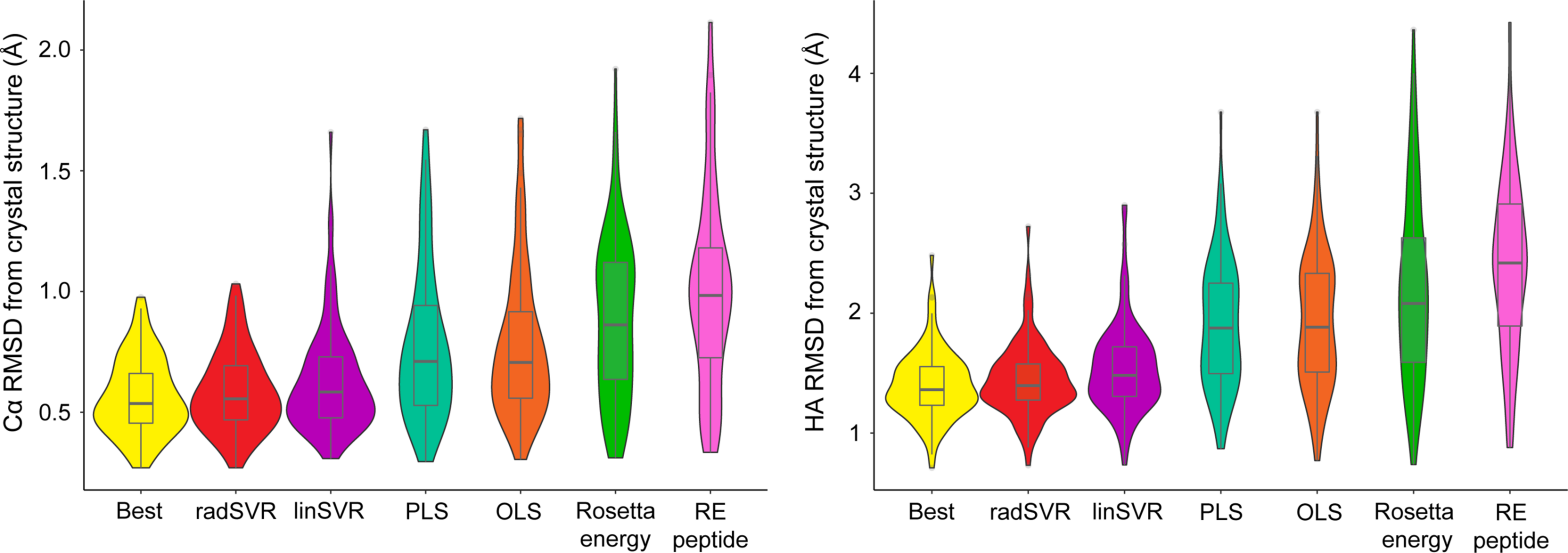
SVR functions outperform least squares functions and energy scores in identifying the best decoy. For each of the structures in **Table S1** (excluding the six test structures), the trained functions were used to select the most optimal decoy from the 500 produced. The best decoy (lowest peptide HA RMSD from structure) was also identified, as were the lowest scoring by total or peptide-only Rosetta energy. These decoys were then used to calculate Cα (left) and HA RMSD (right) from experimental structure, indicated by each violin. Distributions are sorted from left-to-right by ascending mean. The SVR functions clearly outperform other methods of decoy selection (RE peptide, Rosetta energy for the peptide alone). Mirroring the data in **Figure 3**, the two SVR functions were statistically indistinguishable from one another, as well as from the best decoy. Boxes span the first and third quartiles, lines indicate the median.

We used the four trained functions to predict the lowest HA RMSD decoy for each peptide-MHC structure, excluding the six test structures. For comparison, we also selected decoys based on lowest Rosetta energy for the whole peptide-MHC or the peptide alone. For each structure we also again identified the actual best decoy, as measured by lowest HA RMSD from the structure. We then compared these decoys to their crystallographic structures. The decoys selected by SVR functions exhibited Cα/HA RMSD distributions very similar to those for the best decoys (**Figure 4**). In fact, there was no significant difference between RMSDs of the best decoys and those selected by SVR (one-tailed t-test p=0.21), nor was the difference between radSVR and linSVR performance significant (two-tailed t-test p=0.10). In contrast, selection with either OLS or PLS resulted in decoys with significantly higher RMSD, as did selection by total energy or peptide energy. These results highlight that, pending additional improvements to the accuracy of generated decoys (for example, through improved sampling or more accurate energy functions), little improvement in selection accuracy is likely to be found beyond these SVR-based regression functions.

**Figure 5:**
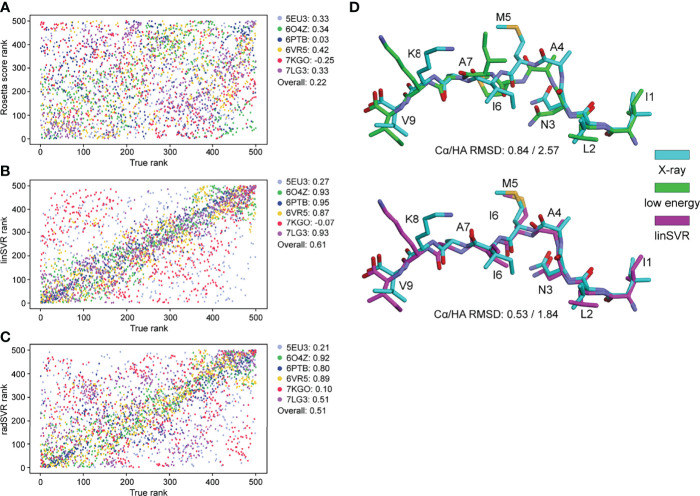
SVR selection functions show improved performance in a non-biased test set. 500 models for six structures not included in training were generated with the ref2015 KIC protocol. All decoys were ranked by peptide HA RMSD from the crystal structure (“true rank”) and compared to the ranking by Rosetta energy **(A)** the linSVR function **(B)** and the radSVR function **(C)**. The legends in **(A–C)** indicate the peptide-MHC PDB ID and the associated Spearman correlation between HA RMSD and Rosetta score or function prediction, as well as the overall correlation. The linSVR function is the strongest performer, ranking four out of six of the structures with high accuracy. A fifth (5EU3) was poorly ranked due to limited sampling around a highly accurate model as discussed in the text. **(D)** Example of performance with 6PTB, comparing the peptide crystallographic coordinates with the decoy with the lowest Rosetta energy (top) and the optimal decoy selected by linSVR (bottom). Cα/HA RMSD values are indicated for each case.

To further validate performance, trained functions were used to select decoys for the six nonameric peptides presented by HLA-A*02:01 whose structures are available but were not included in training: PDB ID 5EU3 (70), 6O4Z (71), 6PTB (20), 6VR5 (72), 7KGO (73), and 7LG3 (74). For each target peptide-MHC, we generated 500 decoys using the same template and ref2015 KIC modeling protocol as above. We then ranked decoys for each target from best (rank #1) to worst (rank #500) by HA RMSD from the crystal structure (the true rank), Rosetta energy (lowest to highest), and the two SVR-based predictive functions. Decoys ranked by Rosetta energy correlated poorly with true decoy rank (**Figure 5A**). However, for 4 of the 6 targets, the SVR-based predictor ranking was highly correlated with the true rank (**Figures 5B, C** **)**. For example, 6PTB ranking by linSVR correlated with the true rank with a Spearman correlation of 0.95, in sharp contrast to a 0.03 correlation between Rosetta energy and true rank. The overall correlation between true rank by HA RMSD and predictor ranks was 0.61 and 0.51 for linSVR and radSVR respectively, compared to 0.22 for Rosetta energy. The improvement of decoy selection by linSVR over Rosetta energy for 6PTB is illustrated in **Figure 5D**, where the top decoy selected by linSVR clearly matches the crystallographic structure better than the lowest energy structure.

Notably, for 5EU3 the correlation between true and predicted rank was poor for both SVR functions. However, when we compared actual to predicted HA RMSD (**Figure S4**), we found the deficiency in ranking was a result of limited sampling around a conformation very close to the crystallographic structure, with all decoys tightly clustered around a HA RMSD of 1.4 Å (i.e., there were no good vs. bad decoys for the functions to discriminate between) (**Figure S4B**). For two of the six targets (7KGO and 7LG3), the predicted HA RMSD values from radSVR were monotonic, despite a high range of actual HA RMSD sampled (**Figure S4C**). This was not seen with linSVR. Together, these results reinforced the accuracy of the SVR functions over Rosetta energy in selecting optimal decoys and suggested further that linSVR is a more appropriate predictor than the radSVR function.

### Application to a Relevant Tumor Neoantigen

**Figure 6:**
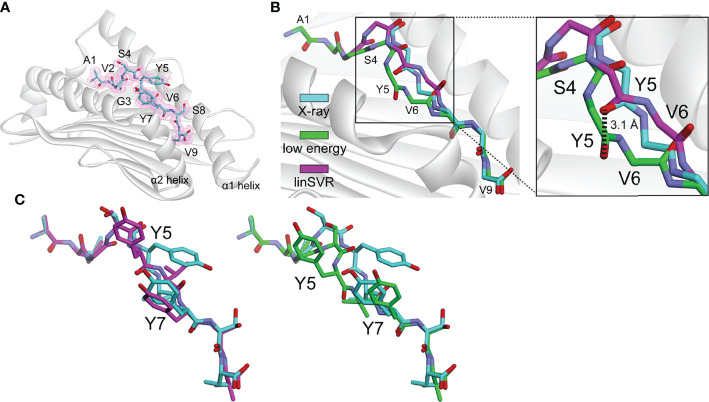
The linSVR function selects a more accurate model for a novel neoantigen structure. **(A)** Structure of the AVGSYVYSV neoantigen bound to HLA-A*02:01, with 2F_o_-F_c_ electron density at 1σ shown. **(B)** Comparison of lowest energy decoy and the linSVR selected decoy for AVGSYVYSV after modeling (peptide backbone shown only). The lowest energy decoy has the backbone incorrectly modeled from Ser4 through Val6, leading to a 3.1 Å displacement in the carbonyl oxygen at Tyr5 as shown in the inset. **(C)** Structure and decoy comparison, showing the entire peptides. The error in the position of the Tyr5 side chain is exacerbated in the low energy decoy. Colors are the same as in panel B.

As a test of our modeling and trained selection approaches, we deployed it against a novel tumor neoantigen whose structure has not yet been reported. The neoantigen AVGSYVYSV was identified in a melanoma patient and shown to induce a T cell response in a healthy donor (75). We crystallized and determined the structure of AVGSYVYSV bound to HLA-A*02:01 at a resolution of 1.9 Å (**Table S3**). The peptide adopted a typical nonameric conformation through the binding groove, with valine at position 6 serving as a secondary anchor and facing down into the groove (**Figure 6A**). We modeled 500 decoys using the ref2015/KIC protocol. The decoy with the lowest Rosetta energy deviated from the actual structure with Cα and HA RMSDs of 0.46 Å and 1.09. The decoy selected by our linSVR function, however, was better, with Cα and HA RMSDs of 0.38 Å and 1.02 Å, respectively (for comparison, the OLS and PLS functions selected a decoy with a HA RMSD of 1.56 Å, and the radSVR function selected a decoy with a HA RMSD of 1.88 Å, all poorer than those selected by either Rosetta energy or linSVR).

A key error in the structure with the lowest Rosetta energy was an incorrect positioning of the peptide backbone from Ser4 to Val6, reflected by a 3.1 Å error in the placement of the Tyr5 carbonyl oxygen (**Figure 6B**). The positions of backbone hydrogen bond donors and acceptors in the centers of peptides have previously been shown to substantially impact TCR binding and T cell sensitivity (39); thus the error in the low energy decoy could be significant. The side chain of Tyr5 of the peptide is incorrectly modeled in both cases, but in the low energy decoy, this error is exacerbated by the error in the backbone (**Figure 6C**). This test demonstrates the applicability of the linSVR function for improving biologically relevant structural predictions.

### Error by Position Reveals the Central Bulges of Peptides Are the Most Challenging to Correctly Model

**Figure 7:**
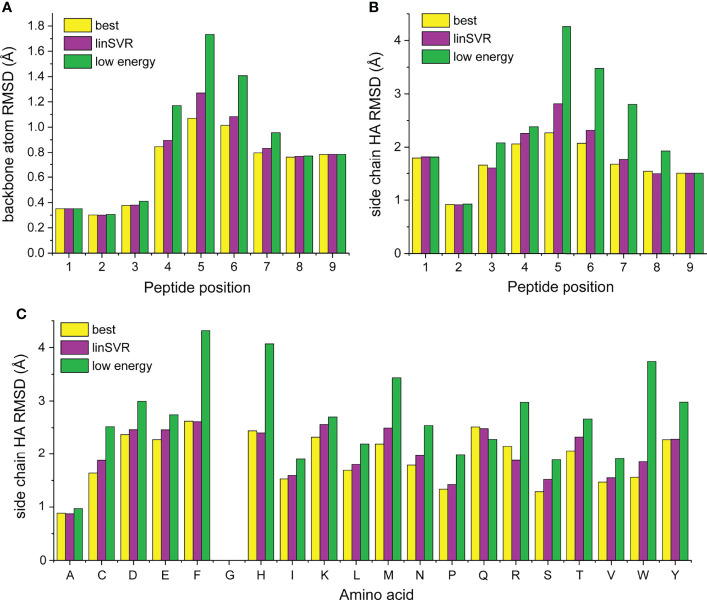
Stratification of peptide RMSD from crystal structure by peptide position and amino acid reveals peptide central bulges are the most difficult to model, without clear trends in amino acid type. **(A, B)** Average RMSDs from crystal structures by peptide position for backbone atoms **(A)** and side chain atoms **(B)**. Data for the best decoys, optimal decoys selected by linSVR, and decoys selected by lowest Rosetta energy are indicated. The central regions of peptides are the most difficult to model correctly. Once again, decoys selected by linSVR are more accurate than those selected by Rosetta energy. **(C)** As in panels **(A, B)**, but heavy atoms by amino acid type. There are no clear trends for modeling accuracy, but selection by Rosetta energy score performs particularly poorly with the large and chemically complex side chains of phenylalanine, histidine, methionine, arginine, tryptophan, and tyrosine.

We next asked how peptide positions and amino acid types were contributing to error in our modeling and selection approaches. For each structure in **Table S1**, we compared the best decoy (lowest HA RMSD from crystal structure), the decoy selected by lowest Rosetta energy, and the best decoy selected by the linSVR function, stratified by amino acid identity at each position of the peptide. Amino acids were only considered if they were represented in three or more peptides at a particular position (for example, tryptophan was present at position 5 in at least three peptides in the structures in **Table S1**). The data are represented in **Figure S5** and compiled into average deviations by peptide position and amino acid in **Figure 7**. Several themes emerge from this data. The best decoy data shows the accuracy and limitations of the ref2015/KIC Rosetta modeling protocol. Generally, the peptide backbone at positions 1-3 can be well modeled (average RMSD < 0.4 Å), while deviations are larger at positions 7-9 (**Figure 7A**; average RMSD near 0.7 Å). Errors in the backbone of the centers of the peptides are higher (average RMSD near 1.0 Å), reflecting the bulges present in nonameric peptides bound to class I MHC proteins. These trends generally hold for side chains, although the range of RMSD is larger, as expected (**Figure 7B**).

Examining the position-dependent deviations by decoy selection method reveals deeper insight and further validates the linSVR selection function (**Figures 7A, B** **)**. Decoys selected by lowest energy are substantially error-prone for positions 4-6 for the backbone (particularly at position 5) and positions 4-7 for the side chains. Decoys selected by linSVR are also error-prone at these positions, but the errors are much smaller. Indeed, the linSVR data are essentially indistinguishable from the best decoy data in many positions, reflecting the agreement seen in **Figure 4**.

Interestingly, the amino acid side chain data do not reveal clear trends by amino acid type (**Figure 7C**). Once again, the linSVR decoy data closely matches the best decoy data. Data for selection by lowest energy is poorer for nearly all amino acids, but particularly so for the large and chemically complex side chains of phenylalanine, histidine, methionine, arginine, tryptophan, and tyrosine.

### Applicability to Other MHC Haplotypes

We last examined the extent to which our modeling and selection processes were generalizable to other HLA haplotypes, despite being trained on data from only HLA-A*02:01. We did not consider different peptide lengths given the template-based structural modeling and the fact that selection models were trained on pockets associated with nonameric peptides. We were substantially limited by the number of non-HLA-A*02:01 structures with nonameric peptides that met our criteria for high resolution and clear peptide electron density. However, in five other HLA-A systems, the linSVR function led to selection of a more accurate decoy than did Rosetta energy in only two cases (**Figure S6**). In 64 HLA-B systems, a more accurate decoy was selected in only 12 cases, and in eight non-classical HLA systems a more accurate decoy was selected in only two cases. Assessments on murine class I MHC proteins were similar, with two of three cases selected more accurately by linSVR for H-2D^d^, three of seven for H-K^b^, and three of eight for H-2K^d^. For H-2D^b^, for which 38 high resolution structures of nonamers were available, linSVR did not select a more accurate decoy for any of them. These results confirm that the selection function trained on HLA-A*02:01 is applicable only to HLA-A*02:01, reflecting the sequence and structural differences among the various class I MHC allotypes and how the regression on structural and energetic terms accounts for unique features of HLA-A*02:01.

## Discussion

There has been long-standing interest in accurate prediction of structural features of peptides bound and presented by class I MHC proteins. This interest has grown with the advent of rapid genome sequencing and the prospect of personalized, peptide-based cancer vaccines, as well as the development of TCR-based molecular and cellular therapeutics. However, while speed and accessibility have improved over the years, improvements in peptide-MHC modeling accuracy have been modest. Accuracy is crucial in peptide-MHC modeling, as TCRs are highly sensitive to subtle perturbations, and small changes in peptide backbone or side chain positions can separate a strong agonist from an irrelevant peptide (15, 76, 77). Here, we explored methods to improve the accuracy of peptide-MHC structural modeling, focusing on nonamers presented by the human class I protein HLA-A*02:01. We addressed a key question common in modeling efforts: from a range of structural models, or decoys, which among them is the closest to the actual three-dimensional structure and thus appropriate to use as a predicted structure?

Modern structural modeling methods typically involve the generation of multiple decoys through various forms of Monte Carlo sampling, frequently using algorithms incorporated into the widely adopted Rosetta modeling suite (41). Most commonly, a final decoy is selected based on the criteria of lowest computed energy. However, across multiple systems, structurally divergent decoys can be very similar in energy, and sometimes the lowest energy decoy is not the most accurate (32–34). While in some cases this could reflect the existence of protein dynamics, a growing consensus is it is more often attributable to inaccuracies and the necessary tradeoffs and simplifications in energy functions (35, 36). Thus, the answer to the question of which decoy is most accurate can be a significant question that, left unaddressed, can lead to errors and uncertainties in structural modeling experiments and the conclusions drawn from them.

Here, we examined the accuracy of Rosetta-based peptide-MHC structural modeling procedures. As has been seen in other cases where high fidelity is required, we show that scoring and selecting decoys by energy is indeed poorly effective at reproducing known structures (35). After identifying a more optimal sampling approach, we used a large database of high-resolution peptide/HLA-A*02:01 structures to train system-specific functions to better predict the most accurate structural model from a set of decoys. The functions included terms from the ref2015 Rosetta energy function used to generate the decoys, weighted for each amino acid of a nonameric peptide and its molecular environment when bound to HLA-A*02:01, as well as general structural features of the peptide-MHC complex. The most accurate functions were implementations of support vector machine regression, which compared to simpler least squares analysis reduces the impact of noise in the data (linSVR) and further allows for more complex relationships among data to be considered (radSVR) (67). The SVR functions identified the most accurate model with extremely high fidelity, with our linSVR function proving the most reliable. Indeed, across a large dataset, the SVR selected decoys were indistinguishable from the best computationally generated structure.

Thus, for nonamers bound by HLA-A*02:01 at least, further improvements can only come from improvements in the actual modeling protocols themselves. Areas for improvement include more accurate energy functions and additional conformational sampling. For the latter, comprehensive sampling of the atoms of the MHC protein could be included: our protocols resulted in no changes to in the backbone and only a 0.21 Å average variation in the side chains of the peptide binding groove. Experimentally, the values are also small (0.48 ± 0.07 Å for the backbone and 1.04 ± 0.08 Å for the side chains), but individual cases can show larger variations that are likely coincident with peptide structural features (49). Allowing select amino acids known to be more conformationally labile (such as the short region linking the short and long components of the class I MHC α2 helix) may lead to further improvements. We might also consider the influence of crystalline environments at cryogenic temperatures, which may limit overall accuracy with energy functions that incorporate other data (78).

Not unexpectedly, the most difficult region of the peptide to model (and the largest contributor to error) is the central bulge that includes positions 4-6 of nonameric peptides. One route to improving modeling in this region could be the incorporation of knowledge-based restraints such as amino acid preferences for secondary anchors and their structural dispositions in peptide/HLA-A*02:01 complexes. Similar restraints are already incorporated into energy functions used in modeling generally, where statistical potentials are used to assess favorability of residue-specific backbone dihedrals and side chain rotamers.

A limitation of our work is the trained decoy selection functions are applicable only to nonamers presented by HLA-A*02:01. On one hand, HLA-A*02:01 represents one of the most common class I MHC proteins in human populations, and nonamers are most frequently presented by HLA-A*02:01 (79). On the other hand, neoantigens or other relevant epitopes are very frequently associated with other HLA proteins. Similarly, peptides of other lengths are relevant for all classical class I MHC proteins. One route past these limitations is to generate additional experimental structural data for other class I MHC proteins and peptide lengths, which could be used for similar training on other alleles and peptide lengths. While not insurmountable, this approach is not practical in the near term. A more rapid route could be inclusion of features describing the variety of MHC residues directly interacting with peptides and developing functions that are either agnostic to or incorporate various peptide lengths, similar to tools for predicting peptide-MHC binding affinity that utilize pseudosequences or gapped alignments (9, 80). As mentioned above, these features could also be treated with knowledge-based restraints or statistical potentials. While these steps await future work, our current results nonetheless serve as an important proof of concept. Importantly, given the significance of nonamer/HLA-A*02:01 complexes, our selection functions (and linSVR in particular) should have immediate utility in select immunotherapeutic and other efforts for which structural information would be advantageous.

## Data Availability Statement

The experimental structural data generated for this study have been deposited into the Protein Data Bank with accession number 7U21. Structural data analyzed are also in the Protein Data Bank with the accession numbers listed in **Table S1**.

## Author Contributions

Database construction, Rosetta modelling, scripting, function training, and statistical analyses were performed by GK. Protein production, crystallization, and structure solution were performed by LW with assistance from GK. Data analysis was performed by all authors. GK and BB conceptualized the project, wrote the manuscript, and secured funding. All authors contributed to the article and approved the submitted version.

## Funding

Supported by National Institutes of Health grant R35GM118166 to BB. GK was supported by a fellowship from the Indiana Clinical and Translational Science Institute, funded by NIH grant UL1TR002529. This work is based upon research conducted at the Northeastern Collaborative Access Team beamlines, which are funded by the National Institute of General Medical Sciences from the National Institutes of Health (P30GM124165). The Eiger 16M detector on the 24-ID-E beam line is funded by a NIH-ORIP HEI grant (S10OD021527). This research used resources of the Advanced Photon Source, a U.S. Department of Energy (DOE) Office of Science User Facility operated for the DOE Office of Science by Argonne National Laboratory under Contract No. DE-AC02-06CH11357.

## Conflict of Interest

The authors declare that the research was conducted in the absence of any commercial or financial relationships that could be construed as a potential conflict of interest.

## Publisher’s Note

All claims expressed in this article are solely those of the authors and do not necessarily represent those of their affiliated organizations, or those of the publisher, the editors and the reviewers. Any product that may be evaluated in this article, or claim that may be made by its manufacturer, is not guaranteed or endorsed by the publisher.
