# Supplementary material for: Physicochemical Heuristics for Identifying High Fidelity, Near-Native Structural Models of Peptide/MHC Complexes
Source: Front Immunol. 2022 Apr 25;13:887759. doi: 10.3389/fimmu.2022.887759 (PMC9084917; doi:10.3389/fimmu.2022.887759)
Supplement: Supplementary file 1 [file DataSheet_1.pdf]

Table S1. Structures used for benchmarking modeling protocols and regression functions. Shaded structures, selected randomly from those not included in our prior work (ref. 20 in the main text), were not included in OLS, PLS, and SVR model training and used instead to validate performance.

PDB	Peptide	PDB	Peptide	PDB	Peptide	PDB	Peptide
1B0G	ALWGFFPVL	2V2X	SLFNTVATL	3MRH	CISGVCWTV	5HHP	GILEFVFTL
1DUZ	LLFGYPVYV	2VLL	GILGFVFTL	3MRI	CINMWCWTV	5HHQ	GIWGFVFTL
1EEY	ILSALVGIV	2X4O	KLTPLCVTL	3MRJ	CINGMCWTV	5MEO	ILGKFLHRL
1EEZ	ILSALVGIL	2X4S	AMDSNTLEL	3MRK	PLFQVPEPV	5MEP	ILGKFLHWL
1I1F	FLKEPVHGV	3D25	VLHDDLLEA	3MRL	CINGVVWTV	5MEQ	ILAKFLHTL
1I1Y	YLKEPVHGV	3FQT	GLLGSPVRA	3MYJ	YMFPNAPYL	5MER	ILAKFLHEL
1I7R	FAPGFFPYL	3FQW	RVASPTSGV	3PWJ	LLYGFVNYV	5N6B	LLWNGPMAV
1I7T	ALWGVFPVL	3FT3	VLHDDLLEA	3PWL	LGYGFVNYI	5NMH	SLYNTIATL
1I7U	ALWGFVPVL	3FT4	VLRDDLLEA	3PWN	LLYGFVNYI	5NMK	SLFNTIAVL
1JHT	ALGIGILTV	3GSO	NLVPMVATV	3QFD	AAGIGILTV	5SWQ	CVNGSCFTV
1QEW	FLWGPRALV	3GSQ	NLVPSVATV	3REW	CLGGLLTMV	5WSH	GVWIRTPTA
1S8D	SLANTVATL	3GSR	NLVPVVATV	3TO2	LACFVLAAV	6EWA	ILKEPVHGV
1S9X	SLLMWITQA	3GSU	NLVPTVATV	3V5H	KVAEIVHFL	6O4Y	KLVVGAVGV
1S9Y	SLLMWITQS	3GSV	NLVPQVATV	3V5K	KVAELVWFL	6O4Z	KLVVVAVGV
1T1W	SLFNTIAVL	3GSW	NLVPMVAAV	4I4W	ILAKFLHRL	6OPD	ILNAMIVKI
1T1X	SLYLTVATL	3GSX	NLVPMVAVV	4K7F	VCWGELMNL	6PTB	ILNAMIAKI
1T1Y	SLYNVVATL	3H7B	MLWGYLQYV	4NNY	RQASLSISV	6PTE	ILNAMITKI
1T1Z	ALYNTAAAL	3HPJ	RMFPNAPYL	4NO5	RQISQDVKL	6SS7	LLWAGPMAV
1T20	SLYNTIATL	3I6G	GLMWLSYFV	5E00	GVWIRTPPA	6SS8	LLWNGPIAV
1TVB	ITDQVPFSV	3KLA	SLLMWITQL	5ENW	GLKEGIPAL	6SSA	LLWNGPMQV
1TVH	IMDQVPFSV	3MR9	NLVPAVATV	5EU3	YLEPGPVTA	6VR1	HMTEVVRRC
2C7U	SLFNTIAVL	3MRB	NLVPMVHTV	5EU4	YLAPGPVTA	6VR5	HMTEVVRHC
2GIT	LLFGKPVYV	3MRC	NLVPMCATV	5EU5	YLEPAPVTA	7KGO	ILLNKHIDA
2GTZ	ALGIGILTV	3MRD	NLVPMGATV	5F9J	YLSPIASPL	7LG2	ALWEIQQVV
2GUO	AAGIGILTV	3MRF	GLCPLVAML	5FA3	GLLPELPAV	7LG3	KLWAQCVQL
2V2W	SLYNTVATL	3MRG	CINGVCWTV	5HHN	GILGLVFTL		

Table S2. Features used for training decoy selection OLS, PLS, and SVR functions. Shaded terms were excluded for some or all peptide positions (as noted) due to zero variance across all decoys.

Per residue energetic/ statistical terms (ref2015)	Brief Description
fa_atr	Inter-residue Lennard Jones attractive potential.
fa_rep	Inter-residue Lennard Jones repulsive potential.
fa_intra_rep	Intra-residue Lennard Jones repulsive potential.
fa_elec	Coulombic potential.
fa_sol	Isotropic Lazaridis-Karplus solvation energy.
lk_ball_wtd	Weighted sum of fa_sol and anisotropic solvation energy correction.
fa_intra_sol_xover4	Intra-residue isotropic Lazaridis-Karplus solvation energy.
hbond_sr_bb	Backbone-backbone hydrogen bonds between residues adjacent in sequence (entirely excluded).
hbond_lr_bb	Backbone-backbone hydrogen bonds between residues distant in sequence (entirely excluded).
hbond_bb_sc	Sidechain-backbone hydrogen bonds.
hbond_sc	Sidechain-sidechain hydrogen bonds (p9 excluded).
rama_prepro	Torsion energy with separate consideration for Pro-X (p1 and p9 excluded).
omega	Probability of backbone dihedral ω (p9 excluded).
p_aa_p	Probability of residue at ϕ/ψ (p1 and p9 excluded).
fa_dun	Internal energy of rotamer à la Dunbrack 2010 smooth backbone-dependent library.
ref	Free energy of an amino acid in denatured state (entirely excluded).
**Structure-derived terms**	
sasa (not from ref2015)	Solvent accessible surface area.
hydro_sasa (not from ref2015)	Hydrophobic solvent accessible surface area.

Table S3. X-ray data and refinement statistics for the AVGSYVYSV/HLA-A*02:01 complex. Values in parenthesis are for the highest resolution shell.

**Data Collection**	
Resolution range (Å)	42.32 - 1.90 (1.97 - 1.90)
Space group	P 1 21 1
Unit cell dimensions (Å)	58.48, 84.65, 84.32
Unit cell angles (°)	90, 90, 90
Unique reflections	61286 (5495)
Completeness (%)	93.43 (85.50)
Mean I/sigma(I)	8.20 (1.49)
R-merge	0.05 (0.21)
R-meas	0.06 (0.30)
R-pim	0.05 (0.21)
CC1/2	1.00 (0.89)
**Refinement**	
Reflections used in refinement	60522 (5495)
Reflections used for R-free	6082 (558)
R-work	0.19 (0.25)
R-free	0.21 (0.27)
Wilson B-factor	16.05
Number of non-hydrogen atoms	7044
macromolecules	6302
ligands	0
solvent	742
Protein residues	768
RMS(bonds)	0.006
RMS(angles)	0.90
Ramachandran favored (%)	97.75
Ramachandran allowed (%)	2.25
Ramachandran outliers (%)	0
Rotamer outliers (%)	1.05
Clashscore	3.92
PDB ID	7U21

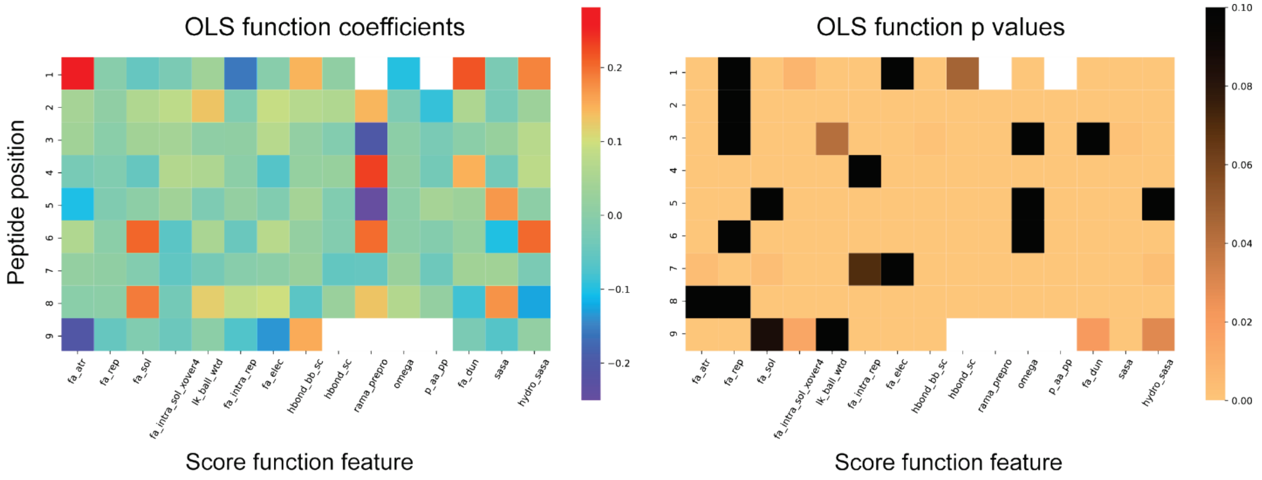

Figure S1. Coefficients (left) and p values (right) for the OLS function trained to predict decoy-to-crystal structure HA RMSD. Empty, non-colored cells correspond to terms which were not included in model training.

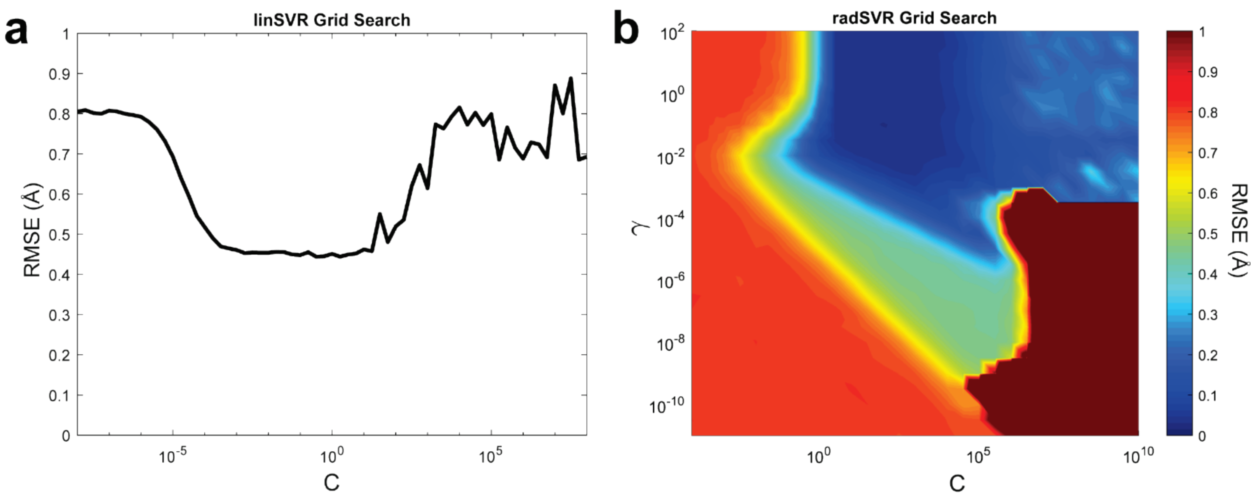

Figure S2. Grid search for SVR hyperparameters illustrating the average 10-fold cross validated RMSE as a function of the regularization parameter *C* and (for radSVR) Gaussian width γ. **A)** *C* for linSVR was selected as $10^{-0.5}$ RMSE=0.4443. **B)** *C* for radSVR was 10^{2}, γ 10^{-1} (RMSE =0.0307). The *z* axis was clipped at 1 to better illustrate the surface at low RMSE hyperparameter combinations (parts of the lower right region have high RMSE in excess of 100).

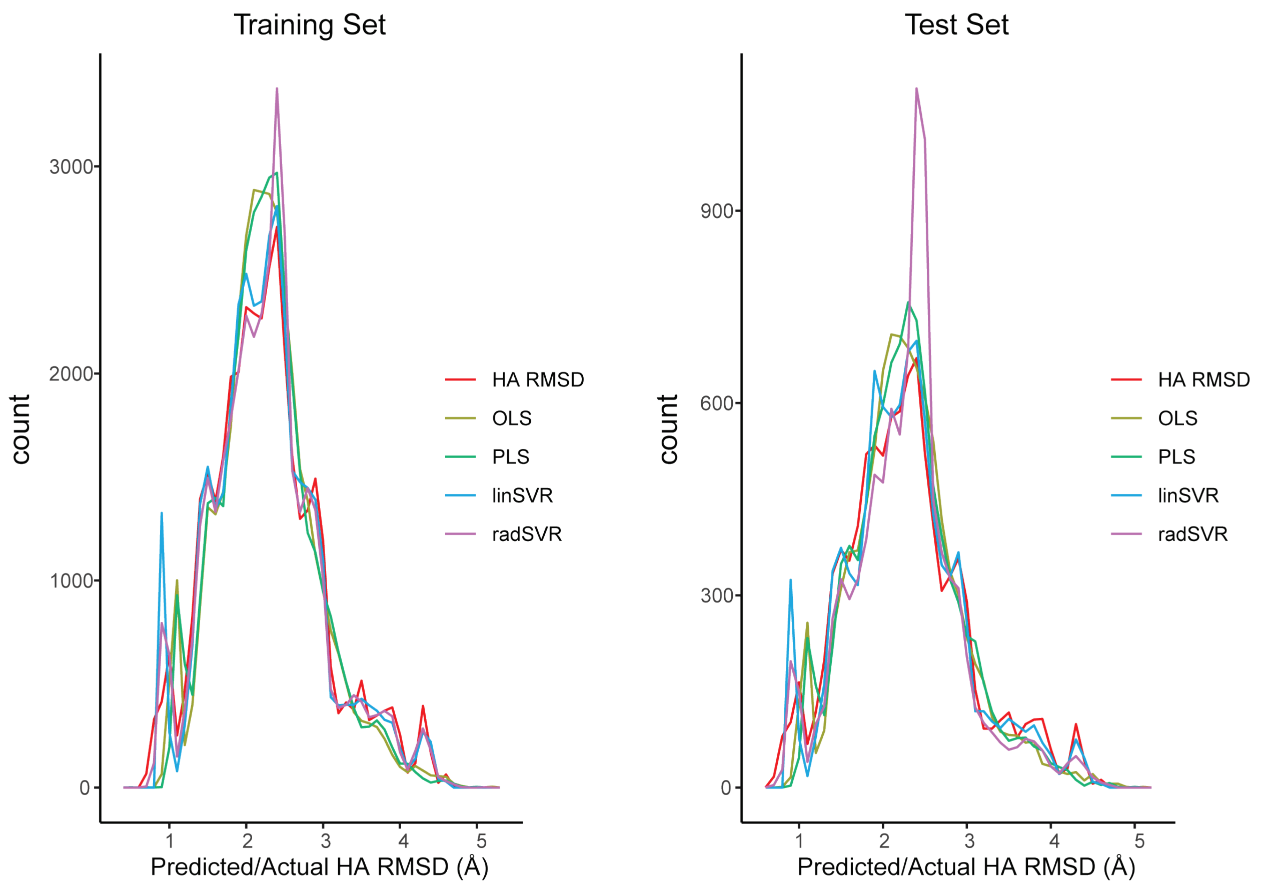

Figure S3. Prediction frequency histograms of trained functions and actual HA RMSD to the training set (left) and test set (right). The OLS and PLS models mistakenly predicted low RMSD (~1.1 and ~2.1 Å) for high RMSD decoys (~3.4-4.5 Å); however, as this behavior was apparent in both the training and test data, it suggests poor model fitting rather than overfitting. The radSVR function erroneously predicted an HA RMSD of 2.5 Å for some decoys regardless of actual RMSD, which was exacerbated in test set predictions and suggests some overfitting in this function as described in the text (see also Fig. 3F).

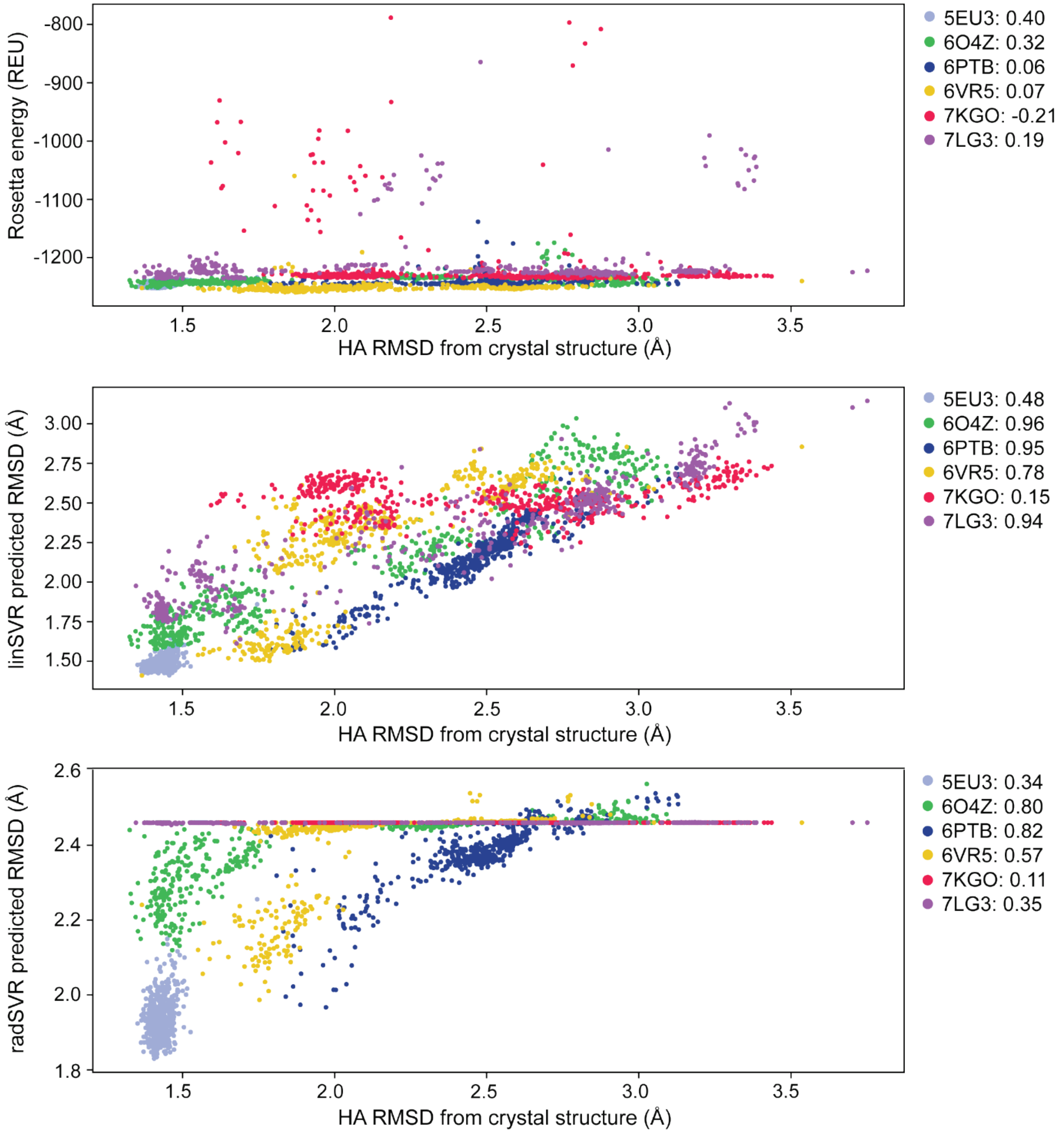

Figure S4. Predicted vs. actual HA RMSD from crystal structure in the non-biased test set for decoys selected by **(A)** lowest Rosetta energy, **(B)** the trained linSVR function, and **(C)** the trained radSVR function. The legends indicate the peptide-MHC PDB ID and the associated Pearson correlation. The linSVR function is again shown to be the strongest performer. The limited sampling for 5EU3 around a very accurate model is clear from the light blue datapoints in panels B and C.

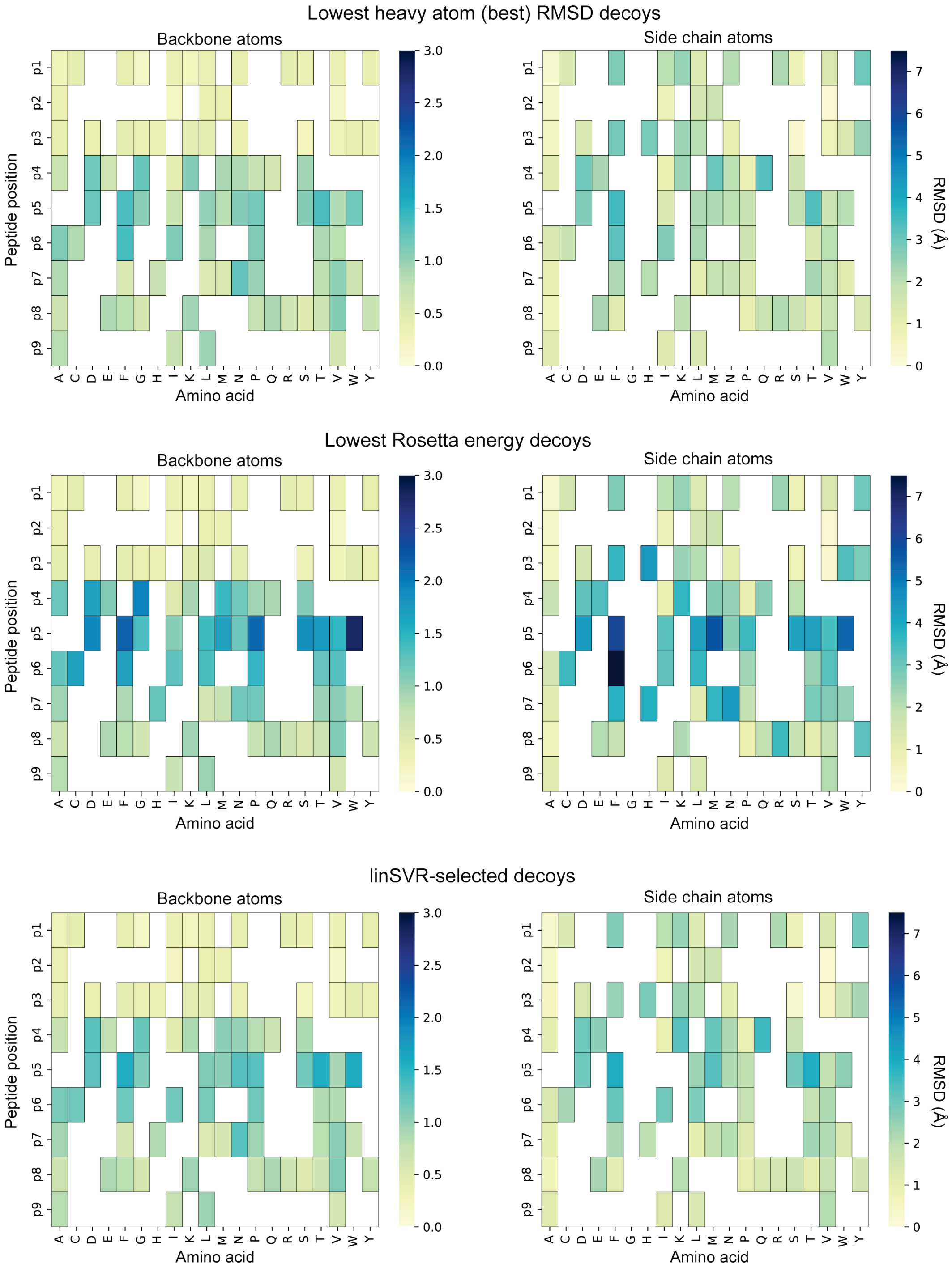

Figure S5. Heat maps for stratification of RMSD from crystal structure by peptide position and amino acid type. Data for the best decoys (lowest HA RMSD from crystal structure) are across the top, the decoy with the lowest Rosetta energy in the middle, and most optimal decoy selected by the linSVR function at the bottom. Backbone atoms are on the left, side chain atoms the right. Amino acids represented in fewer than three structures at a particular position were excluded.

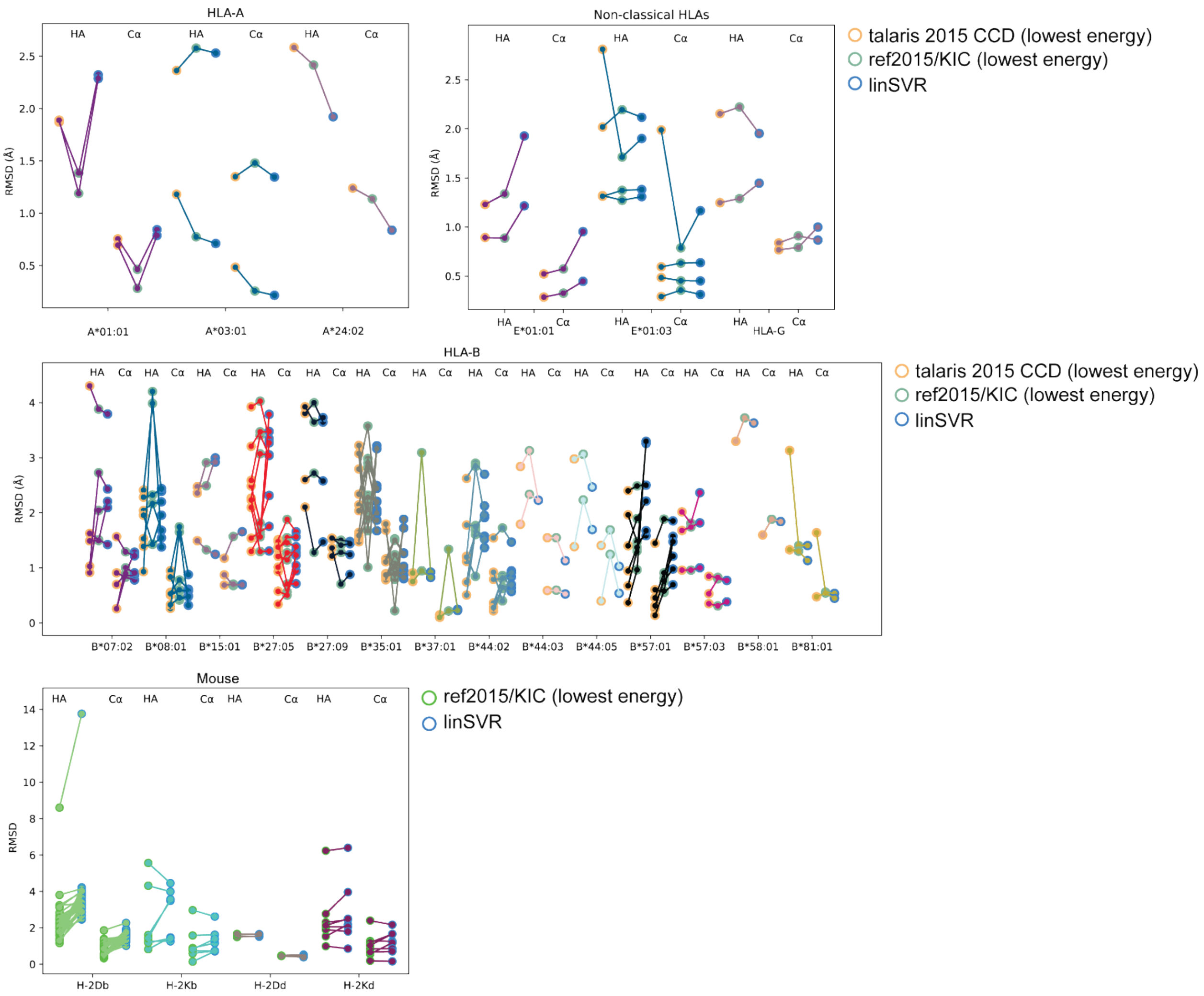

Figure S6. Performance of modeling and selection function with class I MHC proteins other than HLA-A*02:01. Top row shows HLA-A and HLA-E, F, and G in separate panels. Middle row shows HLA-B, and bottom row shows mouse class I. Structures were modeled for instances where high resolution structures with clear electron density exist as described in the text. Plots show RMSD from structures for the peptide heavy atoms (HA) and α carbons (Cα) for each structure of each MHC in adjacent columns. Lines connect RMSD values for decoys with lowest Rosetta energies after modeling with the talaris2014/CCD or ref2015/KIC protocols and the linSVR selected decoy after ref2015/KIC modeling (mouse data at the bottom exclude the talaris2014/CCD data). The stacked lines in each column indicate structures of different peptides bound to the same MHC.
